# Biochemical Characterization of a Disaccharidase From *Enterococcus faecalis* CTB

**DOI:** 10.1002/fsn3.71363

**Published:** 2026-01-26

**Authors:** Yaping Yan, Yajie Li, Wanyi Wang, Wenhui Li, Jing Yang, Xiaodong Han, Zhanying Liu

**Affiliations:** ^1^ School of Chemical Engineering Inner Mongolia University of Technology Hohhot Inner Mongolia P. R. China; ^2^ Engineering Research Center of Inner Mongolia for Green Manufacturing in Biofermentation Industry Hohhot Inner Mongolia P. R. China; ^3^ Specialized Technology Research and Pilot Public Service Platform for Biological Fermentation in Inner Mongolia Hohhot Inner Mongolia P. R. China; ^4^ Inner Mongolia Ecological Environment Scientific Research Institute Limited Hohhot Inner Mongolia P. R. China; ^5^ College of Life Science Inner Mongolia Agricultural University Hohhot P. R. China

**Keywords:** catalytic mechanisms, disaccharidase, *Enterococcus faecalis*, enzyme activity, the optimal substrate

## Abstract

A disaccharidase (GenA) from *Enterococcus faecalis* CTB was heterologously expressed in *Escherichia coli* and purified to > 95% homogeneity using chromatographic techniques. The enzyme exhibited a monomeric molecular weight of 54 kDa and demonstrated hydrolytic activity toward maltose, cellobiose, and lactose, but not sucrose. Kinetic analysis revealed maltose as the preferred substrate (Km = 0.27 ± 0.05 mM, *V*_max_ = 33.8 ± 2.24 μM/min), followed by lactose (Km = 0.42 ± 0.04 mM, *V*_max_ = 42.0 ± 2.91 μM/min) and cellobiose (Km = 0.47 ± 0.06 mM, *V*_max_ = 51.0 ± 1.90 μM/min). GenA also hydrolyzed synthetic substrates including PNP‐α‐D‐glucoside and PNP‐β‐D‐galactopyranoside. The enzyme displayed substrate‐dependent optimal conditions: 40°C–60°C and pH 7.5–9.0. MgCl_2_ enhanced enzymatic activity 2.0–4.0 fold across all substrates, while NiCl_2_ and MnCl_2_ were generally inhibitory. These findings provide insights into GenA's catalytic mechanisms and highlight its potential applications in biocatalysis and industrial biotechnology.

## Graphical abstract

This study explores GenA, a novel disaccharidase from *Enterococcus faecalis* . Cloned/expressed in *Escherichia coli* and 98% purified, GenA hydrolyzes maltose (optimal), cellobiose, lactose, with substrate‐dependent optimal T/pH. MgCl_2_ boosts activity; NiCl_2_/MnCl_2_ inhibit. It aids catalytic mechanism studies and industrial biocatalysis.

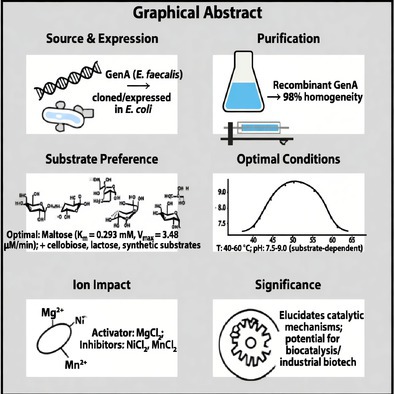

## 1 Introduction

Disaccharides are carbohydrates composed of two monosaccharide units, with significant application value across various industries (Yuan et al. 2018). In the food sector, sucrose is widely used as a sweetener, lactose is crucial in dairy products, and maltose is important in brewing and baking (Fels and Bunzel 2022). Pharmaceutically, disaccharides serve as excipients in drug formulations and as prebiotics (Chen et al. 2022). In biotechnology, they are used to produce glucose syrups and in developing enzyme‐based biosensors (Daneshmand et al. 2019). However, the utilization of disaccharides faces several challenges. Enzymatic hydrolysis can be a limiting factor in some applications, requiring specific disaccharides for efficient breakdown. Digestive issues like lactose intolerance restrict the use of certain disaccharide‐containing products. Stability concerns arise during food processing and storage, with some disaccharides prone to unwanted reactions (Gallo et al. 2024).

Disaccharidases are enzymes that catalyze the hydrolysis of disaccharides into their constituent monosaccharides. These enzymes play a crucial role in carbohydrate digestion and absorption in the human body (Kluch et al. 2020; Viswanathan and Rao 2023). Recent research on disaccharidases has made significant progress. There is growing interest in gut microbial disaccharidases and their interactions with human enzymes (Berding et al. 2016). Metagenomic studies are discovering novel enzymes from diverse microbial communities. Medical applications, including treatments for lactose intolerance, are being explored. New high‐throughput assays are improving enzyme characterization (Cristina Julio‐Gonzalez et al. 2023). Studies on the regulation of intestinal disaccharidase expression by diet and hormones are ongoing (Paquet Luzy et al. 2024). This research is advancing both fundamental understanding and practical applications of disaccharidases in medicine, biotechnology, and industry.

Enzymes have emerged as critical biocatalysts with significant biotechnological potential, particularly marine‐derived enzymes showing exceptional properties for industrial applications, including alkaline phosphatases, thermostable luciferases (Homaei et al. 2013), and cytochrome P450 enzymes (Sharifian et al. 2020). Advanced enzyme immobilization techniques using gold nanorods have enhanced the stability and catalytic performance of proteases like actinidin and papain (Homaei and Etemadipour 2015), while bioluminescent enzyme systems have found increasing use in medical research (Sharifian et al. 2018) and marine enzymes are being classified for diverse industrial applications (Ghattavi and Homaei 2023). Disaccharides, composed of two monosaccharide units, have significant value across food, pharmaceutical, and biotechnology industries, though their utilization faces challenges including enzymatic hydrolysis limitations, digestive issues like lactose intolerance, and stability concerns during processing. Disaccharidases, which hydrolyze disaccharides into constituent monosaccharides, play crucial roles in carbohydrate digestion and absorption, with growing interest in gut microbial disaccharidases and their interactions with human enzymes.

*Enterococcus faecalis* , a probiotic species widely distributed in natural environments and prominent in respiratory and digestive tract microbiota (Badr et al. 2024; Krawczyk et al. 2021), demonstrates metabolic versatility by secreting L‐type lactic acid and converting carbohydrates efficiently. This Gram‐positive bacterium is widely distributed in natural environments, particularly in livestock and poultry habitats, and serves as a prominent member of the gastrointestinal and respiratory tract microbiota in humans and animals. *Enterococcus faecalis* demonstrates remarkable metabolic versatility, secreting L‐type lactic acid and efficiently converting various carbohydrates into lactic acid, which is subsequently absorbed and utilized by the host organism (Wei et al. 2021). The probiotic functions of *E. faecalis* encompass promoting intestinal digestion, maintaining microbial flora balance in the gastrointestinal tract, and enhancing immune system function in both humans and animals, conferring significant value in medical and food industry applications (Tinrat et al. 2018; Han et al. 2024; Huang et al. 2025).

Our research group previously isolated *E. faecalis* CTB, a cellulose‐degrading strain from pig intestines, and in this study, we characterized the biochemical properties of GenA, a disaccharidase from this strain, focusing on its substrate versatility and reaction optimization to address knowledge gaps in understanding disaccharidases from probiotic bacteria and explore their potential applications in biotechnology and medicine. In this study, we characterized a disaccharidase (GenA) from *E. faecalis* CTB and determined its biochemical characteristics. The research aims to fill a gap in our understanding of disaccharidases from probiotic bacteria and explore potential applications in biotechnology and medicine. By characterizing GenA, we seek to elucidate its role in carbohydrate metabolism and its potential for industrial and therapeutic uses.

## 2 Materials and Methods

### 2.1 Molecular Cloning and Heterologous Expression of GenA

The *GenA* gene (MK714072) was amplified from the genome of *E. faecalis* strain CTB by polymerase chain reaction (PCR) using the primer (GenA‐F: TACTTCCAATCCAATGCCATATGTCAATTTTGAAAAATGA, GenA‐R: TTATCCACTTCCAATGCTATTTATAATTCTTCTCC). The amplified PCR product was cloned into the pET30‐TEV/LIC vector (Novagen, USA) using a ligation‐independent cloning (LIC) strategy. This vector incorporates a 6× His tag and a TEV protease cleavage site at the N‐terminus of the inserted gene (Aslanidis and de Jong 1990).

The recombinant plasmid pET30‐*GenA* was transformed into *E. coli* BL21‐Codon Plus (DE3) cells using a heat‐shock procedure. Positive transformants were selected on LB‐agar plates supplemented with 34 μg/mL kanamycin (Sigma‐Aldrich). Selected colonies were inoculated into 5 mL LB medium containing kanamycin and grown for 12 h at 37°C with agitation. The overnight culture was then transferred into 1 L LB medium with kanamycin and grown at 37°C with agitation until the OD_600_ reached 0.6–0.8.

### 2.2 Overexpression and Purification of GenA

Protein expression was induced by adding isopropyl β‐D‐1‐thiogalactopyranoside (IPTG) to a final concentration of 100 μM, followed by incubation at 16°C for 16–18 h. Cells were harvested by centrifugation at 5000×*g* for 15 min at 20°C and resuspended in lysis buffer (25 mM Tris–HCl, pH 8.0, 500 mM NaCl). The cell suspension was lysed by sonication on ice using 2 s pulses with 5 s pauses for a total of 15 min, then centrifuged at 30,000×*g* for 40 min at 4°C.

The supernatant was loaded onto a Ni‐NTA resin column (Qiagen, Germany) pre‐equilibrated with lysis buffer. Non‐specifically bound proteins were removed with washing buffer (25 mM Tris–HCl, pH 8.0, 500 mM NaCl, 50 mM imidazole). The target protein was eluted using elution buffer (25 mM Tris–HCl, pH 8.0, 500 mM NaCl, 100 mM imidazole).

The eluted protein was concentrated to 2 mL using an Amicon Ultra filter (30 kDa molecular‐weight cutoff, Millipore) and diluted to 10 mL with buffer containing 25 mM Tris–HCl, pH 8.0. The protein solution was then applied to a HiTrap Q HP column (GE, USA) and eluted with a linear NaCl gradient (0–1 M).

Peak fractions containing GenA were concentrated to 2 mL and loaded onto a HiLoad 16/60 Superdex 200 pg. column (GE, USA) pre‐equilibrated with 25 mM Tris–HCl, pH 8.0, 200 mM NaCl, and 1 mM DTT. The sharp peak corresponding to GenA was pooled and concentrated to 10 mg/mL using an Amicon Ultra filter.

### 2.3 Characterization of GenA by SDS‐PAGE and Western Blot

The purity and identity of recombinant GenA were assessed using SDS‐PAGE and Western blot analysis. The purified GenA samples were mixed with 5× SDS loading buffer and heated at 95°C for 5 min. The samples were then loaded onto a 12% polyacrylamide gel alongside a molecular weight marker. Electrophoresis was performed at 120 V for 1 h and the gel was stained with Coomassie Brilliant Blue R‐250. The protein concentration was estimated using a NanoDrop 2000 spectrophotometer (Thermo Scientific, USA).

For Western blot analysis, proteins were transferred from the SDS‐PAGE gel to a 0.22 μm PVDF membrane using the Trans‐Blot Turbo Transfer System (Bio‐Rad). The membrane was blocked with 5% skim milk in TBST (Tris‐Buffered Saline, 0.1% Tween‐20) for 1 h at room temperature. The membrane was then incubated with anti‐His‐tag monoclonal antibody (Proteintech) diluted 1:1000 in TBST for 12 h at 4°C. After washing three times with TBST for 5 min each, the membrane was incubated with LICOR IRDye 800CW Goat Anti‐Rabbit IgG secondary antibody (1: 10,000 dilution) for 90 min at room temperature. The membrane was washed again three times with TBST. Protein signals were detected using the Odyssey Clx LICOR imaging system.

### 2.4 Enzymatic Activity Determination and Kinetic Parameter Analysis

The enzymatic activity of GenA was determined by measuring the amount of reducing sugars released from substrates using the dinitrosalicylic acid (DNS) assay (Liao et al. 2017). This method is based on the redox reaction between 3,5‐dinitrosalicylic acid and reducing sugars under alkaline conditions, producing 3‐amino‐5‐nitrosalicylic acid, a reddish‐brown chromophore. The assay involved incubating 0.25 mg of GenA enzyme with 1 mM substrate at 55°C for 30 min, adding 0.5 mL of DNS reagent, heating at 100°C for 5 min, and measuring the absorbance at 540 nm. The color intensity is proportional to the reducing sugar concentration, allowing for quantification of enzyme activity.

A glucose standard curve was prepared using 0–1.0 mL of 1 mg/mL glucose solution, adjusted to 1 mL final volume, mixed with 0.5 mL DNS reagent, heated at 100°C for 5 min, cooled, and adjusted to a final volume of 10 mL. Absorbance was measured at 540 nm. Enzymatic activity was assessed using the following substrates: 4‐nitrophenyl‐α‐D‐galactopyranoside, p‐nitrophenyl‐β‐D‐galactopyranoside, cellobiose, maltose, lactose, and sucrose.

One international unit (IU) of enzymatic activity was defined as the amount of enzyme that releases 1 μmol of reducing sugars (measured as glucose equivalent) per minute under the assay conditions. Kinetic parameters (Km and *V*_max_) were determined by fitting experimental data to the Michaelis–Menten equation using nonlinear regression analysis. Substrate concentrations ranging from 0.1 to 2.0 mM were used for each kinetic analysis. Initial reaction velocities (V) were measured at each substrate concentration and plotted against substrate concentration [S]. The data were fitted to the Michaelis–Menten equation: *V* = (*V*_max_ × [*S*])/(Km + [*S*]) using Graphpad prism software to obtain the kinetic parameters.

### 2.5 Effects of Temperature, pH, and Metal Ions on GenA Activity

The thermodynamic stability of the GenA enzyme was assessed using cellobiose, maltose, and lactose as substrates. Activity measurements were conducted after 15 min incubations at temperatures ranging from 30°C to 70°C, with 5°C intervals. The pH‐dependent activity profile of GenA was investigated across a pH range of 4–11 using three buffer systems: 0.1 M disodium hydrogen phosphate citrate buffer (pH 4–8), 0.05 M Tris–HCl buffer (pH 8–9), and 0.1 M sodium carbonate sodium bicarbonate buffer (pH 9–11). The effects of various metal ions on GenA activity were examined using 5 mM concentrations of MgCl_2_, MnCl_2_, CuCl_2_, BaCl_2_, FeCl_3_, LiCl_2_, CaCl_2_, CoCl_2_, NiCl_2_, and AlCl_3_.

For thermal stability, the GenA enzyme was pre‐incubated at temperatures ranging from 20°C to 100°C for 30 min. For pH stability, the enzyme was tested across pH 2.0–11.0 using appropriate buffer systems for 30 min. And then, the GenA enzyme activity assays were checked using maltose as substrate in 25 mM Tris–HCl buffer with DNS detection at 540 nm, at 40°C and pH 7.5.

## 3 Results

### 3.1 Molecular Cloning and Recombinant Expression of GenA

**FIGURE 1:**
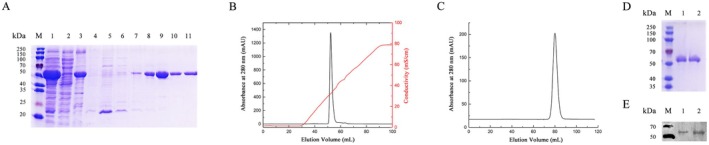
Expression, purification, and identification of recombinant GenA. (A) SDS‐PAGE showing expression of GenA in *E. coli* . M: molecular mass marker (kDa); Lane 1: supernatant fraction of cell lysate; Lane 2: flowing fraction of cell lysate; Lane 3: precipitation fraction of cell lysate; lanes 4–11: Elution buffer containing 0, 10, 25, 50, 100, 150, 200, and 500 mM imidazole, respectively. (B, C) Purification of GenA by ion‐exchange chromatography and size‐exclusion chromatography. (D) GenA protein was purified by a HiLoad 16/60 Superdex‐200 column and checked by SDS‐PAGE. Lane 1–2: the sharp peak from 78 to 85 mL elution volume. (E) Identification of GenA by western blotting. M: molecular mass marker (kDa); Lanes 1 and 2: GenA eluted by 100 and 150 mM imidazole, respectively.

The GenA gene was successfully amplified and cloned into the pET30‐TEV/LIC vector using a ligation‐independent cloning strategy. Recombinant GenA protein was overexpressed in *E. coli* BL21‐Codon Plus (DE3) cells and found predominantly in the soluble fraction of the cell lysate, indicating proper folding and solubility. Initial purification was achieved using immobilized metal affinity chromatography (IMAC) with a Ni‐NTA resin column. The recombinant GenA, featuring a 6 × His tag, was efficiently bound to the column and eluted with 100–200 mM imidazole (Figure 1A).

### 3.2 Purification and Biochemical Characterization of GenA

To obtain GenA protein with uniform surface charge distribution and homogeneity, ion‐exchange chromatography (IEC) and size‐exclusion chromatography (SEC) were employed for further purification after initial Ni‐IMAC purification. At pH 8.0, GenA protein bound almost entirely to the IEC column and was eluted with 300 mM NaCl (Figure 1B). The eluted GenA was then subjected to SEC using a column pre‐equilibrated with 25 mM Tris–HCl (pH 8.0), 300 mM NaCl, and 1 mM DTT. A sharp peak was detected between 75 and 85 mL elution volume, corresponding to a molecular weight of approximately 50 kDa, suggesting that GenA exists as a monomer in solution (Figure 1C).

SDS‐PAGE analysis revealed an observed molecular mass of ~54 kDa, consistent with the calculated molecular weight. The purity of GenA was estimated to be greater than 95% (Figure 1D). Western blot analysis using an anti‐His‐tag antibody confirmed the identity of the purified GenA protein, showing a single stained band (Figure 1E). These purification steps resulted in highly pure, homogeneous GenA protein suitable for subsequent biochemical characterization and activity assays.

### 3.3 Substrate Specificity and Kinetic Analysis

**FIGURE 2:**
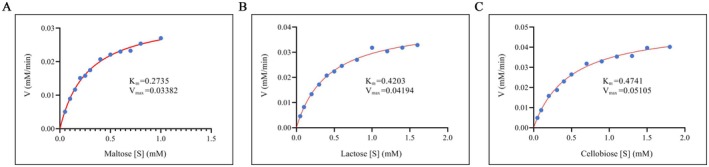
Michaelis–Menten kinetic curves of GenA against different substrates: (A) Michaelis–Menten kinetic curve of GenA with maltose as substrate. (B) Michaelis–Menten kinetic curve of GenA with lactose as substrate. (C) Michaelis–Menten kinetic curve of GenA with cellobiose as substrate.

The disaccharide hydrolase activity of GenA was assessed using four disaccharides: maltose, cellobiose, lactose, and sucrose. Kinetic parameters were determined using Lineweaver‐Burk double‐reciprocal plots. Results showed that GenA exhibited hydrolytic activity toward maltose, cellobiose, and lactose, but not sucrose. Maltose emerged as the optimal substrate for GenA, with a Km of 0.27 mM and a *V*_max_ of 33.8 μM/min (Figure 2A). Lactose was the second‐best substrate with a Km of 0.42 mM and a *V*_max_ of 41.9 μM/min (Figure 2B). Cellobiose was also hydrolyzed by GenA with a Km of 0.47 mM and a *V*_max_ of 51.0 μM/min (Figure 2C).

To further explore the catalytic versatility of GenA, its activity was tested on synthetic substrates. The enzyme demonstrated hydrolytic activity against PNP‐α‐D‐glucoside, PNP‐α‐D‐galactopyranoside, and PNP‐cellobioside. This substrate promiscuity suggests that GenA possesses a broad catalytic repertoire, capable of hydrolyzing structurally diverse carbohydrate moieties (Table 1). The ability of GenA to hydrolyze multiple glycosidic bonds indicates a potentially flexible active site or multiple catalytic domains. These findings highlight GenA's functional versatility and its potential for various biotechnological applications. However, further research, including detailed kinetic analyses and structural studies, is necessary to fully characterize GenA's catalytic properties and explore its potential applications in biocatalysis and industrial biotechnology.

**TABLE 1:** Kinetic analysis of the GenA.

Substrate	Km (mM)	*V*_max_ (μM/min)
Maltose	0.27 ± 0.05	33.8 ± 2.24
Lactose	0.42 ± 0.04	42.0 ± 2.91
Cellobiose	0.47 ± 0.06	51.0 ± 1.90
Sucrose	—	—
PNP‐α‐D‐glucoside	0.30 ± 0.32	19.7 ± 3.31
PNP‐α‐D‐galactopyranoside	0.34 ± 0.47	16.4 ± 1.91
PNP‐cellobioside	0.49 ± 0.05	29.7 ± 1.72
PNP‐β‐D‐glucopyranoside	—	—
PNP‐β‐D‐glucopyranoside	—	—
Salicyloside	—	—

*Note:* Values shown are mean ± SD, calculated from three replicates.

### 3.4 The Optimal Temperature and pH of GenA Enzyme

**FIGURE 3:**
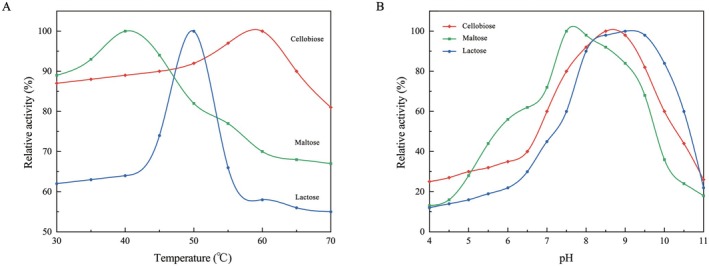
Effect of temperature and pH on GenA activity. (A) Effects of different temperatures on the activity of GenA enzyme on substrates. (B) Effects of pH on the activity of GenA enzyme on substrates. Maltose (green), lactose (blue) and cellobiose (red).

The GenA enzyme exhibited substrate‐dependent thermal and pH optima, as determined by DNS assay. The catalytic activity of GenA was set to 1 at the optimal temperature, with relative activities at different temperatures. The optimal temperatures were found to be 50°C for lactose, 40°C for maltose, and 60°C for cellobiose (Figure 3A). This 20°C range suggests significant thermal flexibility in GenA's structure and function, indicating complex enzyme‐substrate interactions that influence both thermal stability and catalytic efficiency.

The pH‐dependent activity profile of GenA was investigated using lactose, maltose, and cellobiose as substrates across a pH range of 4–11. Results revealed substrate‐specific pH optima: lactose at pH 8.5, maltose at pH 7.5, and cellobiose between pH 8.0–9.0 (Figure 3B). These findings indicate GenA's preference for slightly alkaline conditions, with optimal activity in the neutral to mildly basic range. The observed substrate‐dependent pH optima suggest nuanced enzyme‐substrate interactions and potential conformational changes in the enzyme's active site under varying pH conditions. Notably, cellobiose exhibited a broader optimal pH range, indicating enhanced pH tolerance with this substrate.

The GenA enzyme demonstrates variable thermal and pH stability characteristics based on experimental analysis. Thermal stability assessment reveals that GenA maintains 100% relative activity across a moderate temperature range from 20°C to 60°C, with significant activity decline at 70°C. The enzyme retains 80% of its original activity, followed by a steep decrease to 65% at 80°C, 30% at 90°C, and only 12% residual activity at 100°C. This thermal profile indicates that GenA exhibits good thermostability up to 60°C but experiences rapid denaturation beyond this threshold. pH stability analysis demonstrates that GenA shows optimal stability at neutral pH 7, with decreased performance under both acidic and basic conditions. The enzyme exhibits moderate tolerance to slightly alkaline conditions, maintaining 98% activity at pH 8 and 96% at pH 9, but shows greater sensitivity to acidic environments with activities of 97% at pH 6, 89% at pH 5, 69% at pH 4, and 73% at pH 3 (Table 2).

**TABLE 2:** Thermal and pH stability of GenA enzyme.

Treated with temperature (°C)	Relative activity (%)	Treated with pH	Relative activity (%)
20	100	3	73
30	100	4	69
40	100	5	89
50	100	6	97
60	100	7	100
70	80	8	96
80	65	9	98
90	30	10	94
100	12	11	65

### 3.5 Metal Ion Effects on Enzymatic Activity of GenA

**FIGURE 4:**
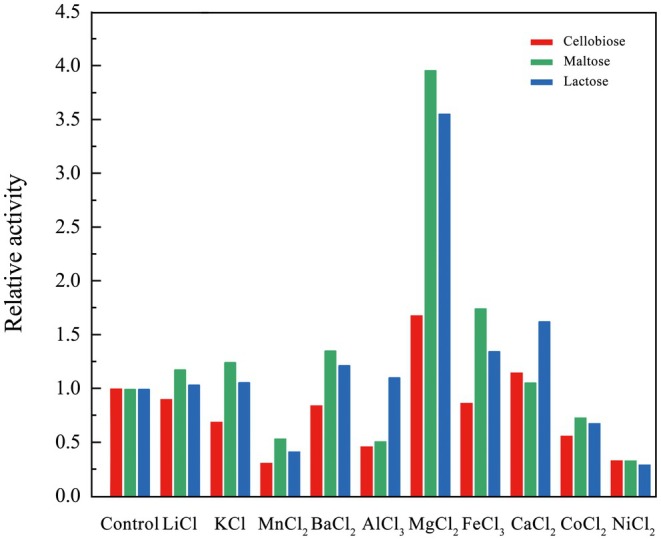
Effects of mental oins on GenA enzyme activity. Control: 25 mM Tris–HCl, pH 8.0, 200 mM NaCl. Maltose (green), lactose (blue), and cellobiose (red).

The effects of various metal ions on the catalytic activity of GenA enzyme were determined using cellobiose, maltose, and lactose as substrates. The results revealed substrate‐specific patterns of activation and inhibition. For cellobiose, MgCl_2_ demonstrated the most pronounced activation, while MnCl_2_ and NiCl_2_ exhibited strong inhibitory effects. In the case of maltose, MgCl_2_ and FeCl_3_ induced significant activation, with MgCl_2_ enhancing activity by a factor of four. Conversely, NiCl_2_, MnCl_2_, and AlCl_3_ elicited inhibitory responses. For lactose, MgCl_2_ and CaCl_2_ manifested strong activation, with MgCl_2_ augmenting activity 3.5‐fold, while NiCl_2_ and MnCl_2_ displayed inhibitory properties (Figure 4). Notably, MgCl_2_ consistently exhibited a potent activating effect across all substrates, whereas NiCl_2_ and MnCl_2_ generally suppressed GenA activity. These observations underscore the intricate interplay between metal ions, enzymatic activity, and substrate specificity, providing valuable insights into the catalytic mechanisms of GenA.

## 4 Discussion

This study presents the successful overexpression, purification, and biochemical characterization of GenA, a disaccharidase from *E. faecalis* . The recombinant GenA was expressed in a prokaryotic system and purified to 98% homogeneity using a series of chromatographic techniques, as confirmed by SDS‐PAGE and western blot analysis. GenA exhibited hydrolytic activity toward maltose, cellobiose, and lactose, but not sucrose, demonstrating a selective substrate specificity profile. The observed kinetic parameters revealed maltose as the optimal substrate, followed by lactose and cellobiose. The differential substrate affinities reflect fundamental enzyme‐substrate interaction mechanisms, where the lower Km value for maltose indicates higher substrate affinity due to optimal geometric complementarity between maltose's α‐1,4‐glucosidic linkage and GenA's active site architecture.

The absence of activity toward sucrose provides mechanistic insights into GenA's substrate recognition. Sucrose contains an α‐1,2‐glycosidic bond between glucose and fructose, which requires different spatial orientation compared to the β‐1,4 (cellobiose, lactose) and α‐1,4 (maltose) bonds that GenA can accommodate. This suggests that GenA's active site architecture has evolved specifically to recognize and bind β‐1,4 and α‐1,4 linkages but cannot properly position sucrose for catalytic hydrolysis. The demonstration of activity against synthetic substrates (PNP‐α‐D‐glucoside, PNP‐α‐D‐galactopyranoside, and PNP‐cellobioside) indicates remarkable catalytic promiscuity and active site flexibility. This substrate versatility suggests that GenA possesses conformational plasticity allowing accommodation of diverse substrates while maintaining the core hydrolysis mechanism, highlighting its potential for biotechnological applications requiring broad substrate specificity.

GenA exhibited substrate‐dependent optimal temperatures and pH levels, revealing complex enzyme‐substrate interactions that influence both thermal stability and catalytic efficiency through substrate‐induced conformational dynamics. The temperature‐dependent optima reflect substrate‐specific enzyme‐substrate complex stability, where maltose's α‐1,4 linkage induces tighter interactions that become thermally labile at higher temperatures, while cellobiose's β‐1,4 linkage requires higher thermal energy to achieve optimal catalytic conformation, indicating that different substrates stabilize distinct enzyme conformational states with varying thermal stabilities. Simultaneously, the substrate‐dependent pH optima provide insights into the protonation states of catalytic residues (likely histidine, aspartate, and glutamate), where varying pH requirements suggest that different substrates alter the pKa values of active site residues through substrate‐induced conformational changes, requiring specific protonation states for optimal nucleophilic activation of water during glycosidic bond hydrolysis.

The metal ion studies revealed contrasting effects where Mg^2+^ consistently enhanced GenA activity across all substrates while Ni^2+^ and Mn^2+^ exhibited inhibitory effects, reflecting fundamental differences in metal cofactor mechanisms. The universal activation by Mg^2+^ indicates multiple mechanistic roles including direct catalytic involvement through coordination with catalytic residues and water molecules to enhance nucleophilicity during glycosidic bond hydrolysis, structural stabilization by bridging enzyme‐substrate interactions, and conformational optimization through favorable active site geometry changes. Conversely, the inhibitory effects of Ni^2+^ and Mn^2+^ result from their inappropriate coordination geometry and electronic properties compared to Mg^2+^, leading to displacement of essential Mg^2+^ without equivalent catalytic function and formation of catalytically incompetent enzyme forms where different coordination preferences and Lewis acidity distort active site architecture and disrupt optimal charge distribution required for efficient catalysis.

In conclusion, this comprehensive characterization of GenA reveals a sophisticated biocatalyst with evolved mechanisms for efficient carbohydrate metabolism in probiotic bacteria. The enzyme's versatility, substrate‐dependent properties, and metal ion modulation capabilities make it a promising candidate for further development in biotechnology and industrial applications. Future research should focus on structural studies to elucidate the molecular basis for substrate‐dependent properties, enzyme engineering to enhance specific characteristics, and evaluation of performance in relevant industrial processes to fully exploit GenA's biotechnological potential.

## Author Contributions

**Yaping Yan:** investigation (equal), supervision (equal). **Yajie Li:** supervision (equal), writing – original draft (equal), writing – review and editing (equal). **Wanyi Wang:** visualization (equal). **Wenhui Li:** conceptualization (equal). **Jing Yang:** conceptualization (equal), software (equal). **Xiaodong Han:** data curation (equal), investigation (equal), methodology (equal), project administration (equal), validation (equal), writing – original draft (equal). **Zhanying Liu:** formal analysis (equal), funding acquisition (equal), resources (equal), validation (equal).

## Conflicts of Interest

The authors declare no conflicts of interest.

## Data Availability

All datasets generated and/or analyzed during the current study are fully included in this published article.
